# Use of European registries to characterise the changing landscape of disease progression and treatment of spinal muscular atrophy (SMA): opportunities, pitfalls and challenges

**DOI:** 10.1186/s13023-026-04491-3

**Published:** 2026-08-05

**Authors:** Nicolas Deltour, Seung Yeon Lee, Elizabeth M. Garry, Liza R. Gibbs, Shane McElwee, Renee M. Sajedian, Emmanuelle Jacquot, Siyana Kurteva, Anabel Ferreras, Annie Poll, Ben Porter, Rhian Davies, Jack Kennedy, Maggie C. Walter, Simone Thiele, Marlène Jagut, Marjan Cosyns, Liesbeth de Waele, Nicolas Deconinck, Jana Haberlová, Lenka Mokrá, Maria Grazia Cattinari, Eduardo F. Tizzano, Chiara Marini-Bettolo, Lindsay Murphy, Anne-Berit Ekström, Miriam Rodrigues, Anna Ambrosini, Victoria Hodgkinson, Michela Guglieri, Mencia de Lemus Belmonte, Kieran C. Breen, Laurent Servais, The Study Investigators Group, Aurore Daron, Stephanie Delstanche, Laura Vanden Brande, Alain Maertens de Noordhout, Stéphanie Paquay, Van den Bergh, Vinciane Van Parys, Nicolas Dubuisson, Gauthier Remiche, Florence Christiaens, Jonathan Baets, Alicia Alonso-Jimenez, Diane Beysen, Véronique Bissay, Nathalie Smeets, Karine Pelc, Katrien De Braekeleer, Jan De Bleecker, Arnaud Vanlander, Sarah Herdewyn, Kristl Claeys, Geertrui Peirens, Philip Van Damme, Anna-Karin Kroksmark, Olesja Parmová, Lenka Juříková, Kryštof Prášil, Neil Bennett

**Affiliations:** 1 Aetion Iberia, S.L., Barcelona, Spain; 2 TREAT-NMD Services Ltd., Newcastle upon Tyne, UK; 3 Aetion, Inc, New York, NY USA; 4 Freelancer, France, France; 5 University of British Columbia, Vancouver, Canada; 6 Health Innovation North East and North Cumbria, Newcastle upon Tyne, UK; 7 Jumping Rivers Ltd, Newcastle upon Tyne, UK; 8 Friedrich-Baur-Institute at the Department of Neurology, University Hospital, LMU Munich, Germany; 9 Sciensano, Brussels, Belgium; 10 University Hospitals Leuven, Leuven, Belgium; 11 Queen Fabiola Children’s University Hospital, Brussels, Belgium; 12 Pediatric Neurology, University Hospital in Motol, Prague, Czech Republic; 13 Institut biostatistiky a analýz s.r.o, Brno, Czech Republic; 14 FundAME, Barcelona, Spain; 15 The John Walton Muscular Dystrophy Research Centre, Translational and Clinical Research Institute, Newcastle University and Newcastle Hospitals NHS Foundation Trust, Newcastle upon Tyne, UK; 16 Department of Pediatrics, Institute of Clinical Sciences, University of Gothenburg. Queen Silvia Children’s Hospital, Sahlgrenska University Hospital, Gothenburg, Sweden; 17 Neurology, Te Whatu Ora - Auckland, Auckland, New Zealand; 18 Fondazione Telethon, Milan, Italy; 19 University of Calgary, Calgary, Canada; 20 St Andrew’s Healthcare, Barcelona, UK; 21 Paediatric Neuromuscular Diseases, University of Oxford, Northampton, UK; 22 Paediatric Neuromuscular Diseases, University of Oxford, Oxford, UK

**Keywords:** Spinal muscular atrophy (SMA), Rare disease, Registries, Real-world evidence, Pediatric/paediatric, Neuromuscular disease (NMD), Neurodegenerative, Disease modifying treatment (DMT)

## Abstract

**Background:**

Spinal muscular atrophy (SMA), a rare neurodegenerative disorder with an estimated prevalence of 1 in 14,300 live births and is the leading genetic cause of mortality in infants and children. Since the approval of new disease-modifying treatments (DMTs; nusinersen (Spinraza) in 2017, onasemnogene abeparvovec-xioi (Zolgensma) in 2020, and oral risdiplam (Evrysdi) in 2021), studies have reported changes in disease progression. This retrospective cohort study analyzed six SMA registries within the TREAT-NMD network, selected via feasibility assessment. These registries comprise data from nine European countries: three clinician-based registries (Belgium, Czech Republic plus Slovakia, Sweden) and three patient-based registries (Germany plus Austria, Spain, United Kingdom plus Ireland) covering the period spanning April 2008 and May 2023.

**Results:**

Among 2,188 SMA patients with genetically confirmed 5q SMA, the most common SMA subtype was type 2 (SMA2; *n* = 914, 41.8%) followed by types 3 (SMA3; *n* = 779, 35.6%) and 1 (SMA1; *n* = 432, 19.7%). Treatment with at least one DMT was reported among 1,321 (60.4%) of patients and increased over time; nusinersen was the most common DMT (*N* = 1,003; 75.9%) followed by risdiplam (*N* = 403; 30.5%) and onasemnogene (*N* = 101; 7.6%). Among treated patients with SMA1, SMA2, and SMA3, best functional status reported was “sitter” for 36.6%, 60.9%, and 5.3%, and “walker” for 12.0%, 24.6%, and 87.8%, respectively. For SMA1 and SMA2, best motor milestone reported was “sit without support” for 27.2% and 38.0%, and “roll onto side” for 18.5% and 2.0%; for SMA3, “climb stairs” was reported for 63.4% and “walk 10 metres without assistance” reported for 19.3%. Missingness of functional status and motor milestone among patients with SMA1, SMA2, and SMA3 was lower among those treated (27.9%, 8.5%, and 5.5%) as accounted for almost all never treated (100%, 97.8%, and 100%).

**Conclusions:**

Functional status and motor milestones were well captured after treatment but rarely reported before treatment or in patients who were never treated, limiting evaluation of treatment related changes. Areas of improvement for registry data quality have been identified to reduce data missingness, increase standardisation, and consequently enhance their ability to inform regulatory decision making.

**Supplementary Information:**

The online version contains supplementary material available at https://doi.org/10.1186/s13023-026-04491-3.

## Background

Spinal muscular atrophy (SMA) is a rare progressive neurodegenerative disorder with an estimated prevalence of 1 in 14,300 live births, and it is the leading genetic cause of mortality in infants and children [1–3]. SMA is caused by mutations in the survival motor neuron 1 gene, which results in progressive loss of alpha motor neurons, severe muscle weakness, and can result in early death [4, 5]. The absence of SMN1 can be partially compensated by SMN2 which is very similar to SMN1- but produces most of time a truncated non-functional protein. A higher number of SMN2 copies result in a milder phenotype.

SMA encompasses heterogenous phenotypes of motor impairment and related comorbidities and is categorised into groups defined according to age of symptom onset and maximum motor function achieved (type 1,2,3,4 and presymptomatic). SMA type 1 (SMA1) is the most common disease type and is associated with severe manifestations of disease including severe hypotonia and respiratory and swallowing difficulties. Patients with SMA1 are diagnosed prior to six months of age, are typically unable to sit independently, and have a typical life expectancy of two years without treatment. Patients with SMA type 2 (SMA2) are diagnosed between six and 18 months of age, are able to sit but not walk, and typically experience a shortened lifespan. Patients with type 3 (SMA3) and type 4 (SMA4) are diagnosed in childhood and adulthood, respectively, and have typical survival probabilities within the first 60 years of life. In addition to these types, some patients are classified separately as being presymptomatically treated, according to age of diagnosis and treatment [5–7].

In 2017, the European Medicines Agency (EMA) approved the first disease modifying therapy (DMT) for SMA, nusinersen (Spinraza®) for intrathecal administration [8]. The intravenous gene therapy onasemnogene abeparvovec-xioi (Zolgensma®) was fully approved in 2022 after a conditional approval in 2020 [9, 10], and oral risdiplam (Evrysdi®) was approved in 2021 [5, 11]. All three medications are indicated for treatment of 5q SMA, with precise target populations varying by patient SMA type, *SMN1* mutation type, and number of *SMN2* copies [12]. Since the approval of these DMTs for SMA, studies have reported disease trajectories which differ from the known natural history of SMA, particularly when treatment is initiated early.

To provide broader and longer-term data collection compared to clinical trials, real-world data collections efforts have been initiated in recent years [13–15]. However, even multinational registries [13, 14] often lack sufficient patient numbers to assess disease trajectories in rare phenotypes or to answer specific questions, such as prognosis factor.

Given the changing treatment landscape for SMA, this study leveraged six registries across Europe and aimed to describe the disease progression of patients with SMA, overall and by treatment status and disease type, and to assess treatment utilization over time. We also report on the challenges and limitations of such an approach.

## Methods

This non-interventional retrospective descriptive cohort study characterises patients with SMA from European registries within the TREAT-NMD federated global registry network. The six country-based registries (representing a total of nine countries) selected for this study were identified via a structured fit-for-purpose assessment of 16 registries in the TREAT-NMD network. The questionnaires were based on EMA guidelines for registry-based studies [16], the REQueST tool items [17], FAIR principles [18], and the Structured Process to Identify Fit-For-Purpose Data (SPIFD) [19]. The questions addressed data reliability, relevance, and accessibility related to key data elements identified from the study protocol, including select patient demographics, SMA diagnosis data, DMT use, and post-diagnostic outcomes. Responses to the questionnaires addressed the percentage of patients within each registry with at least one record related to each key data element. Registries were selected for this study based on availability of key data, data quality processes in place, registry size, and time to data acquisition for use in this study.

**Fig. 1:**
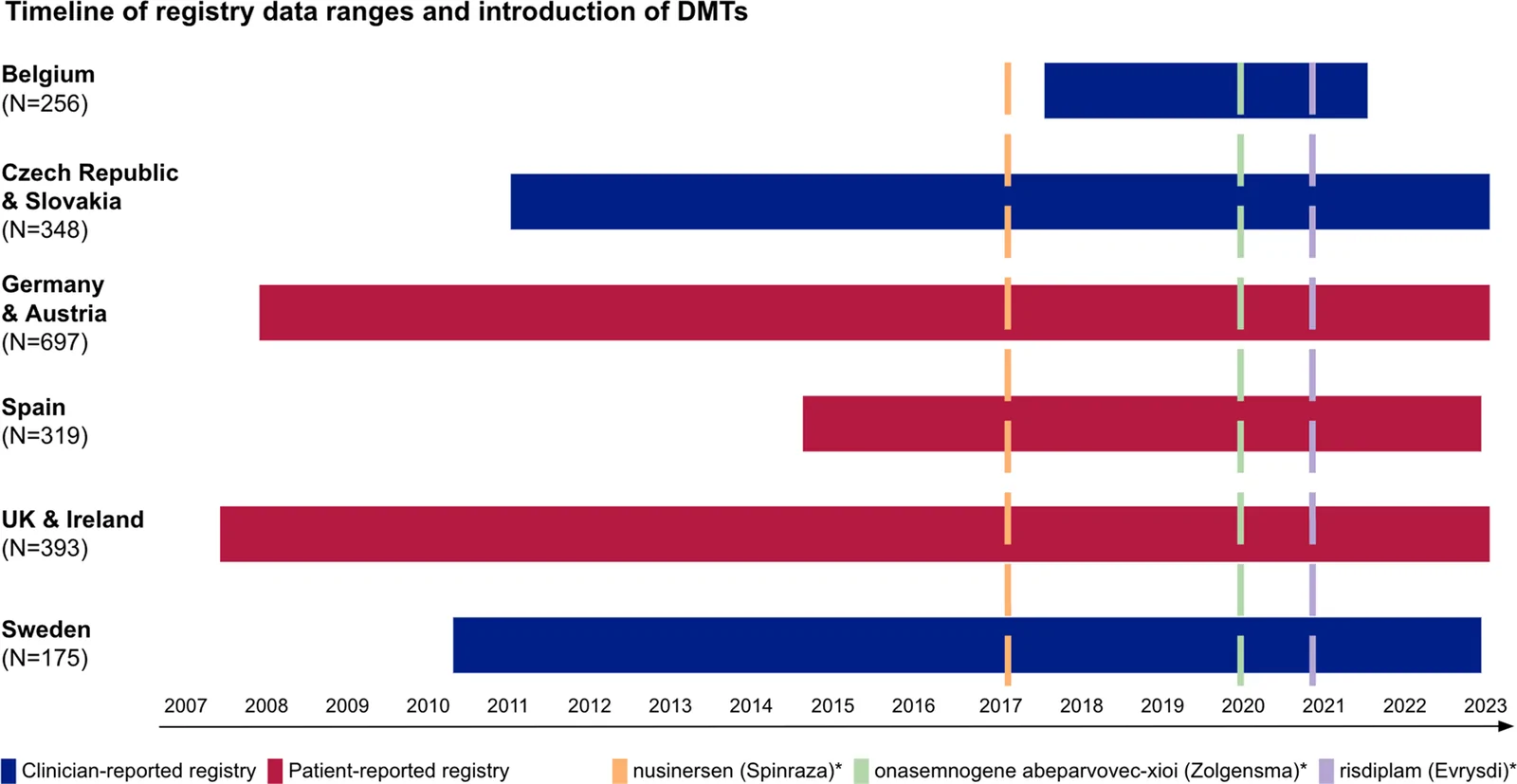
Date ranges of registry data capture and DMT EMA marketing authorisation dates. Legend: *EMA marketing authorisation date; DMT = disease modifying treatment; EMA = European Medicines Agency

Three of the six selected registries were clinician reported: Belgium (data capture from January 2018 through December 2021), Sweden (October 2010 - April 2023), and Czech Republic plus Slovakia (May 2011 - May 2023); the remaining three were patient reported: Spain (February 2015 - April 2023), Germany plus Austria (Apr 2008 - May 2023), and the United Kingdom (UK) plus Ireland (December 2007 - May 2023) (Fig. 1).

The study population comprised all patients with a genetically confirmed 5q SMA diagnosis from the six registries enrolled between the corresponding registry start date (as early as December 2007 in the UK plus Ireland) and end of data availability for this study (as late as May 2023 in the UK plus Ireland, Czech Republic plus Slovakia, and Germany plus Austria). Among this overall cohort of patients, two sub-cohorts were identified based on treatment status: treated patients (who had any exposure to a DMT with a non-missing treatment start date) and never-treated patients (who never had treatment reported in the registry). Additionally, patients were categorised by SMA subtype (SMA1, SMA 2, SMA 3, SMA 4, other, presymptomatic), according to the chronologically most recent type of SMA recorded for a given individual.

Patients were followed prospectively from their entry into the registry until the earliest of the end of data availability in the registry, death, or loss to follow-up. Additionally, historical patient data were captured retrospectively at baseline registration, and thus events occurring prior to patient registration were also captured when available.

Patients were described overall, by treatment status, and by SMA type with respect to age at symptom onset, sex, recorded death, length of follow-up, duration of disease (from symptom onset to the earliest of death, end of data, or loss to follow-up), genetic testing methodologies and results, and treatment utilisation (overall and by medication type). Disease status and progression were described as the best functional status recorded before and after treatment, best motor milestone recorded before and after treatment, and event-free survival time (defined as time from birth until death or permanent ventilation). Treatment patterns were described with respect to treatment type and time to treatment initiation (defined as the time from genetic report date to the first treatment administration date), overall and by calendar time to evaluate changes in care over time. Where applicable, the number and percent of patients with missing data were reported.

## Results

A total of 2,188 patients across the six included registries met study eligibility criteria for the overall cohort (Table 1). Among these patients, 779 were from clinician-reported registries (35.6%; Belgium, Czech Republic plus Slovakia, and Sweden) and 1,409 were from patient-reported registries (64.4%; Germany plus Austria, Spain, and UK plus Ireland). The average duration of follow-up ranged from 51.6 months (Czech Republic plus Slovakia) to 96.9 months (Sweden). Among patients in the overall cohort, the proportion treated with at least one DMT ranged from 43.3% (Germany plus Austria and UK plus Ireland) to 82.1% (Spain). The distribution of SMA types also varied by registry; SMA1 was most commonly reported in the Czech Republic plus Slovakia registry (27.6%), SMA2 was most commonly reported in Spain (48.0%), and SMA3 was most commonly reported in Sweden (40.6%). Data missingness was notable across key variables, including reason for genetic testing (55.7% missing), method of genetic testing (74.3% missing), age at symptom onset (31.0% missing) and disease duration (derived based on symptom onset date, missing for many patients). Missingness for best achieved functional status was 34.5% overall and ranged from 2.7% (Belgium) to 53.7% (UK and Ireland).

**Table 1:** Characteristics of all patients with SMA, overall and by registry

	All (*N* = 2188)	Belgium (*N* = 256)	**Czech Republic & Slovakia** (*N* = 348)	**Germany & Austria** (*N* = 697)	Spain (*N* = 319)	UK & Ireland (*N* = 393)	Sweden (*N* = 175)
**Calendar year of registry entry; n (%)**							
2008	27 (1.2%)	0 (0.0%)	0 (0.0%)	*	0 (0.0%)	23 (5.9%)	0 (0.0%)
2009	23 (1.1%)	0 (0.0%)	0 (0.0%)	13 (1.9%)	0 (0.0%)	10 (2.5%)	0 (0.0%)
2010	41 (1.9%)	0 (0.0%)	0 (0.0%)	8 (1.1%)	0 (0.0%)	*	29 (16.6%)
2011	59 (2.7%)	0 (0.0%)	12 (3.4%)	11 (1.6%)	0 (0.0%)	7 (1.8%)	29 (16.6%)
2012	147 (6.7%)	0 (0.0%)	22 (6.3%)	117 (16.8%)	0 (0.0%)	*	6 (3.4%)
2013	101 (4.6%)	0 (0.0%)	10 (2.9%)	83 (11.9%)	0 (0.0%)	*	7 (4.0%)
2014	203 (9.3%)	0 (0.0%)	16 (4.6%)	123 (17.6%)	0 (0.0%)	48 (12.2%)	16 (9.1%)
2015	187 (8.5%)	0 (0.0%)	16 (4.6%)	75 (10.8%)	7 (2.2%)	85 (21.6%)	*
2016	136 (6.2%)	0 (0.0%)	16 (4.6%)	51 (7.3%)	25 (7.8%)	36 (9.2%)	8 (4.6%)
2017	199 (9.1%)	0 (0.0%)	23 (6.6%)	68 (9.8%)	80 (25.1%)	21 (5.3%)	7 (4.0%)
2018	428 (19.6%)	190 (74.2%)	47 (13.5%)	55 (7.9%)	85 (26.6%)	33 (8.4%)	18 (10.3%)
2019	193 (8.8%)	38 (14.8%)	31 (8.9%)	36 (5.2%)	44 (13.8%)	24 (6.1%)	20 (11.4%)
2020	150 (6.9%)	15 (5.9%)	60 (17.2%)	24 (3.4%)	33 (10.3%)	9 (2.3%)	9 (5.1%)
2021	114 (5.2%)	11 (4.3%)	47 (13.5%)	13 (1.9%)	20 (6.3%)	11 (2.8%)	12 (6.9%)
2022	121 (5.5%)	0 (0.0%)	34 (9.8%)	12 (1.7%)	21 (6.6%)	46 (11.7%)	8 (4.6%)
2023	43 (2.0%)	0 (0.0%)	*	*	*	31 (7.9%)	*
Missing	16 (0.7%)	*	10 (2.9%)	*	0 (0.0%)	*	*
**Female Sex; n (%)**	1059 (48.4%)	129 (50.4%)	176 (50.6%)	341 (48.9%)	145 (45.5%)	179 (45.5%)	89 (50.9%)
**Class of age at symptom onset; n (%)**							
Presymptomatic	8 (0.4%)	0 (0.0%)	6 (1.7%)	*	0 (0.0%)	*	0 (0.0%)
Prenatal	6 (0.3%)	*	0 (0.0%)	*	0 (0.0%)	*	0 (0.0%)
<1 month	32 (1.5%)	*	6 (1.7%)	10 (1.4%)	7 (2.2%)	5 (1.3%)	*
[1 - 3 months)	111 (5.1%)	13 (5.1%)	34 (9.8%)	14 (2.0%)	30 (9.4%)	7 (1.8%)	13 (7.4%)
[3 - 6 months)	146 (6.7%)	20 (7.8%)	38 (10.9%)	24 (3.4%)	31 (9.7%)	18 (4.6%)	15 (8.6%)
[6 - 18 months)	645 (29.5%)	88 (34.4%)	110 (31.6%)	153 (22.0%)	154 (48.3%)	88 (22.4%)	52 (29.7%)
[1.5 - 2 years)	116 (5.3%)	18 (7.0%)	20 (5.7%)	18 (2.6%)	26 (8.2%)	14 (3.6%)	20 (11.4%)
[2 - 6 years)	254 (11.6%)	35 (13.7%)	56 (16.1%)	71 (10.2%)	34 (10.7%)	35 (8.9%)	23 (13.1%)
[6 - 11 years)	59 (2.7%)	10 (3.9%)	18 (5.2%)	11 (1.6%)	6 (1.9%)	7 (1.8%)	7 (4.0%)
[11 - 18 years)	102 (4.7%)	15 (5.9%)	15 (4.3%)	40 (5.7%)	13 (4.1%)	11 (2.8%)	8 (4.6%)
18 years +	30 (1.4%)	*	12 (3.4%)	7 (1.0%)	*	*	*
Missing	679 (31.0%)	48 (18.8%)	33 (9.5%)	347 (49.8%)	16 (5.0%)	202 (51.4%)	33 (18.9%)
**Duration of follow up (months),** Mean (SD)	70.9 (38.4)	55.5 (13.7)	51.6 (34.3)	86.9 (35.4)	58.3 (22.2)	67.8 (43.9)	96.9 (49.9)
**Duration of follow up (months),** Missing; n (%)	16 (0.7%)	*	10 (2.9%)	*	0 (0.0%)	*	*
**Duration of SMA (months),** Mean (SD)	252.1 (195.8)	235.8 (163.3)	209.8 (170.3)	300.7 (215.2)	232 (186)	311.1 (225.9)	211.5 (171.6)
**Duration of SMA (months),** Missing; n (%)	699 (31.9%)	52 (20.3%)	45 (12.9%)	348 (49.9%)	16 (5.0%)	205 (52.2%)	33 (18.9%)
SMA type; n (%)							
SMA Type 1	432 (19.7%)	34 (13.3%)	96 (27.6%)	134 (19.2%)	71 (22.3%)	69 (17.6%)	28 (16.0%)
SMA Type 2	914 (41.8%)	101 (39.5%)	117 (33.6%)	291 (41.8%)	153 (48.0%)	183 (46.6%)	69 (39.4%)
SMA Type 3	779 (35.6%)	103 (40.2%)	120 (34.5%)	269 (38.6%)	93 (29.2%)	123 (31.3%)	71 (40.6%)
SMA Type 4	22 (1.0%)	8 (3.1%)	8 (2.3%)	2 (0.3%)	2 (0.6%)	0 (0.0%)	2 (1.1%)
SMA Type missing	26 (1.2%)	7 (2.7%)	0 (0.0%)	0 (0.0%)	0 (0.0%)	17 (4.3%)	2 (1.1%)
SMA Type other	7 (0.3%)	3 (1.2%)	1 (0.3%)	0 (0.0%)	0 (0.0%)	0 (0.0%)	3 (1.7%)
SMA Type presymptomatic	8 (0.4%)	0 (0.0%)	6 (1.7%)	1 (0.1%)	0 (0.0%)	1 (0.3%)	0 (0.0%)
**Methods used for genetic testing; n (%)**							
DNA Sequencing	0 (0.0%)	*	0 (0.0%)	0 (0.0%)	0 (0.0%)	0 (0.0%)	0 (0.0%)
HRM	0 (0.0%)	*	0 (0.0%)	0 (0.0%)	0 (0.0%)	0 (0.0%)	0 (0.0%)
MLPA	527 (24.1%)	201 (78.5%)	93 (26.7%)	82 (11.8%)	0 (0.0%)	27 (6.9%)	124 (70.9%)
RFLP	5 (0.2%)	*	0 (0.0%)	0 (0.0%)	0 (0.0%)	*	0 (0.0%)
ddPCR	*	*	0 (0.0%)	0 (0.0%)	0 (0.0%)	0 (0.0%)	0 (0.0%)
qRT-PCR	25 (1.1%)	15 (5.9%)	*	6 (0.9%)	0 (0.0%)	*	0 (0.0%)
Missing	1625 (74.3%)	30 (11.7%)	254 (73.0%)	609 (87.4%)	319 (100.0%)	362 (92.1%)	51 (29.1%)
**Patients in treated group; n (%)**	1321 (60.4%)	202 (78.9%)	269 (77.3%)	302 (43.3%)	262 (82.1%)	170 (43.3%)	116 (66.3%)
**Patients in never treated group; n (%)**	847 (38.7%)	54 (21.1%)	79 (22.7%)	380 (54.5%)	57 (17.9%)	218 (55.5%)	59 (33.7%)
**Best achieved functional status; n (%)**							
Non-sitter	123 (5.6%)	11 (4.3%)	44 (12.6%)	30 (4.3%)	11 (3.4%)	18 (4.6%)	9 (5.1%)
Sitter	554 (25.3%)	107 (41.8%)	122 (35.1%)	111 (15.9%)	116 (36.4%)	61 (15.5%)	37 (21.1%)
Walker	757 (34.6%)	131 (51.2%)	96 (27.6%)	210 (30.1%)	163 (51.1%)	103 (26.2%)	54 (30.9%)
Missing	754 (34.5%)	7 (2.7%)	86 (24.7%)	346 (49.6%)	29 (9.1%)	211 (53.7%)	75 (42.9%)

ddPCR = droplet digital polymerase chain reaction; HRM = high resolution melting; IQR = interquartile range; MLPA = multiplex ligation-dependent probe amplification; qRT-PCR = quantitative real-time reverse-transcription polymerase chain reaction; RFLP = restriction fragment length polymorphism; SD = standard deviation; SMA = spinal muscular atrophy; UK = United Kingdom

Note: * indicates *n* < 5 patients

Among the overall cohort, the most common SMA subtype was SMA2 (*n* = 914, 41.8%), followed by SMA3 (*n* = 779, 35.6%) and SMA1 (*n* = 432, 19.7%). Other types were much less frequent, as only 1.2% of patients had SMA4, 0.4 were presymptomatic and 0.3% had SMA0. Across all registries, 26 patients (1.2%) were missing a recorded SMA type. Type was missing for 26 patients. Nearly half of patients were female (48.4%). Overall, the median (interquartile range, IQR) age at symptom onset was 2 months [1, 5] for patients with SMA1 (*N* = 284), 10 months [7, 13] for SMA2 (*N* = 618), and 2.7 years (1.5, 7.0) for SMA3 (*N* = 571) among patients with this information captured. Age was missing for 31.0% of patients (Table 2). The average duration of disease among those with a date of symptom onset reported was 21.0 years, and increased as expected by SMA type (SMA1: 7.5 years, SMA2: 20.0 years, and SMA3: 28.6 years) (Table 3).

**Table 2:** Characteristics of all patients with SMA, overall, by treatment status, and by SMA type

	Overall	By Treatment Status		By SMA Type		
	All (*N* = 2188)	Never-treated (*N* = 847)	Treated (*N* = 1321)^a^	SMA Type 1 (*N* = 432)	SMA Type 2 (*N* = 914)	SMA Type 3 (*N* = 779)
**Registry; n (%)**						
Belgium	256 (11.7%)	54 (6.4%)	202 (15.3%)	34 (7.9%)	101 (11.1%)	103 (13.2%)
Czech Republic & Slovakia	348 (15.9%)	79 (9.3%)	269 (20.4%)	96 (22.2%)	117 (12.8%)	120 (15.4%)
Germany & Austria	697 (31.9%)	380 (44.9%)	302 (22.9%)	134 (31.0%)	291 (31.8%)	269 (34.5%)
Spain	319 (14.6%)	57 (6.7%)	262 (19.8%)	71 (16.4%)	153 (16.7%)	93 (11.9%)
UK & Ireland	393 (20.0%)	218 (25.7%)	170 (12.9%)	69 (16.0%)	183 (20.0%)	123 (15.8%)
Sweden	175 (8.0%)	59 (7.0%)	116 (8.8%)	28 (6.5%)	69 (7.5%)	71 (9.1%)
**Sex (% female / % male)**	48.4% / 51.6%	48.6% / 51.4%	48.3% / 51.7%	47.7% / 52.3%	50.0% / 50.0%	47.1% / 52.9%
**Age at symptom onset (years)**						
Mean (SD)	2.8 (5.2)	4.4 [7]	2.4 (4.7)	0.3 (0.8)	0.9 (0.8)	5.3 (5.9)
Median [IQR]	1 [0.5, 2]	1.5 [0.8, 4]	1 [0.5, 2]	0.2 [0.1, 0.4]	0.8 [0.6, 1.1]	2.7 [1.5, 7]
Missing; n (%)	683, 31.4%	572 (67.5%)	120 (9.1%)	146 (34.3%)	296 (32.5%)	208 (26.8%)
**SMA type; n (%)**						
SMA Type 1	432 (19.7%)	154 (18.2%)	276 (20.9%)	432 (100.0%)	0 (0.0%)	0 (0.0%)
SMA Type 2	914 (41.8%)	361 (42.6%)	540 (40.9%)	0 (0.0%)	914 (100.0%)	0 (0.0%)
SMA Type 3	779 (35.6%)	299 (35.3%)	476 (36.0%)	0 (0.0%)	0 (0.0%)	779 (100.0%)
SMA Type 4	22 (1.0%)	13 (1.5%)	9 (0.7%)	0 (0.0%)	0 (0.0%)	0 (0.0%)
SMA Type missing	26 (1.2%)	16 (1.8%)	9 (0.7%)	0 (0.0%)	0 (0.0%)	0 (0.0%)
SMA type other	7 (0.3%)	*	4 (0.3%)	0 (0.0%)	0 (0.0%)	0 (0.0%)
SMA Type presymptomatic	8 (0.4%)	*	7 (0.5%)	0 (0.0%)	0 (0.0%)	0 (0.0%)
**DMTs; n (%)**						
Patients treated with at least one DMT^a^	1321 (60.4%)	-	1321* (100.0%)	276 (63.9%)	540 (59.1%)	476 (61.1%)
Patients treated at least once with nusinersen	1003 (45.8%)	-	1003 (75.9%)	215 (49.8%)	363 (39.7%)	411 (52.8%)
Patients treated at least once with onasemnogene	101 (4.6%)	-	101 (7.6%)	68 (15.7%)	22 (2.4%)	4 (0.5%)
Patients treated at least once with risdiplam	403 (18.5%)	-	403 (30.5%)	64 (14.8%)	226 (24.7%)	101 (13.0%)

DMT = disease modifying treatment; IQR = interquartile range; SD = standard deviation; SMA = spinal muscular atrophy; UK = United Kingdom

^a^1321 patients have been considered as TREATED with at least one DMT and with no missing DMT intake start date (of note, 20 patients have been excluded as DMT intake start date was not available)

Note: * indicates *n* < 5 patients

**Table 3:** Disease characteristics of all patients with SMA, overall and by SMA types 1, 2, and 3

	Overall (*N* = 2188)	SMA Type 1 (*N* = 432)	SMA Type 2 (*N* = 914)	SMA Type 3 (*N* = 779)
**Class of age at symptom onset; n (%)**				
Presymptomatic	8 (0.4%)	0 (0.0%)	0 (0.0%)	0 (0.0%)
Prenatal	6 (0.3%)	*	*	0 (0.0%)
<1 month	32 (1.5%)	22 (5.1%)	*	5 (0.6%)
1 - <3 months	111 (5.1%)	102 (23.6%)	7 (0.8%)	*
3 - <6 months	146 (6.7%)	108 (25.0%)	36 (3.9%)	*
6 - <18 months	645 (29.5%)	47 (10.9%)	500 (54.7%)	95 (12.2%)
1.5 - <2 years	116 (5.3%)	*	47 (5.1%)	68 (8.7%)
2 - <6 years	254 (11.6%)	0 (0.0%)	18 (2.0%)	236 (30.3%)
6 - <11 years	59 (2.7%)	0 (0.0%)	*	54 (6.9%)
11 - <18 years	102 (4.7%)	*	*	94 (12.1%)
≥18 years	30 (1.4%)	0 (0.0%)	0 (0.0%)	16 (2.1%)
Missing; n (%)	679 (31.0%)	148 (34.3%)	296 (32.4%)	208 (26.7%)
**Duration of follow up (months),** Mean (SD)	70.9 (38.4)	59.2 (33.2)	76.4 (38.4)	73.1 (38.6)
**Duration of follow up (months),** Missing; n (%)	16 (0.7%)	7 (1.6%)	*	*
**Duration of SMA (months),** Mean (SD)	252.1 (195.8)	90.1 (78)	240 (166.6)	343.1 (210.2)
**Duration of SMA (months),** Missing; n (%)	699 (31.9%)	153 (35.4%)	299 (32.7%)	210 (27.0%)
**Number of SMN2 copies; n (%)**				
0	15 (0.7%)	*	7 (0.8%)	6 (0.8%)
1	10 (0.5%)	*	*	*
2	321 (14.7%)	235 (54.4%)	52 (5.7%)	29 (3.7%)
3	800 (36.6%)	85 (19.7%)	479 (52.4%)	223 (28.6%)
4	251 (11.5%)	-	30 (3.3%)	208 (26.7%)
>4	10 (0.5%)	*	*	7 (0.9%)
Other	52 (2.4%)	*	*	39 (5.0%)
Missing	729 (33.3%)	105 (24.3%)	337 (36.9%)	263 (33.8%)
**Methods used for genetic testing; n (%)**				
DNA Sequencing	0 (0.0%)	0 (0.0%)	*	0 (0.0%)
HRM	0 (0.0%)	0 (0.0%)	0 (0.0%)	*
MLPA	527 (24.1%)	88 (20.4%)	195 (21.3%)	215 (27.6%)
RFLP	5 (0.2%)	0 (0.0%)	5 (0.5%)	0 (0.0%)
ddPCR	*	0 (0.0%)	*	*
qRT-PCR	25 (1.1%)	*	7 (0.8%)	13 (1.7%)
Missing	1625 (74.3%)	340 (78.7%)	705 (77.1%)	547 (70.2%)
**Reason for genetic testing; n (%)**				
Family screening	38 (1.7%)	5 (1.2%)	9 (1.0%)	19 (2.4%)
Newborn screening	20 (0.9%)	8 (1.9%)	0 (0.0%)	0 (0%)
No screening	902 (41.5%)	155 (36.4%)	381 (41.8%)	347 (44.7%)
Prenatal screening	*	0 (0.0%)	0 (0.0%)	*
Missing	1210 (55.7%)	258 (60.6%)	521 (57.2%)	409 (52.6%)

ddPCR = droplet digital polymerase chain reaction; HRM = high resolution melting; IQR = interquartile range; MLPA = multiplex ligation-dependent probe amplification; qRT-PCR = quantitative real-time reverse-transcription polymerase chain reaction; RFLP = restriction fragment length polymorphism; SD = standard deviation; SMA = spinal muscular atrophy

Note: * indicates *n* < 5 patients

The method used for genetic testing across registries was missing in 74.3% of patients. Fewer than 3% of patients had a screening-related reason for genetic testing reported with 41.5% reporting no screening and 55.7% having no information regarding reason for genetic testing. The low prevalence of presymptomatic patients and newborn genetic testing was not unexpected considering that implementation of newborn screening programs are relatively recent, e.g., 2021 in Germany, Belgium, and Austria, starting in 2023 in Sweden, and pilot programs starting in 2022 in the Czech Republic, Slovakia, select regions of Spain, and the UK [20–23].

A total of 1,321 patients (60.4%) had a record of treatment with at least one DMT with a non-missing DMT initiation date (treated group), and 847 patients (38.7%) had no record of DMT treatment across their available registry data (never treated group). The proportion of patients treated with at least one DMT varied across registries, ranging from 43.3% (Germany plus Austria, UK plus Ireland) to 82.1% (Spain). Among those treated across all SMA types, the most common DMT was nusinersen (*N* = 1,003; 75.9%), followed by risdiplam (*N* = 403; 30.5%) and onasemnogene (*N* = 101; 7.6%), respectively. The distribution of patients treated with any DMT was similar for each SMA type, ranging from 59.1% in SMA2 to 63.9% in SMA1. Nusinersen was the most common treatment for all three types. The distribution of risdiplam and onasemnogene was similar for patients with SMA1 (14.8% vs 15.7%), but risdiplam was more common than onasemnogene for patients with SMA2 (24.7% vs 2.4%) and SMA3 (13.0% vs 0.5%).

**Fig. 2:**
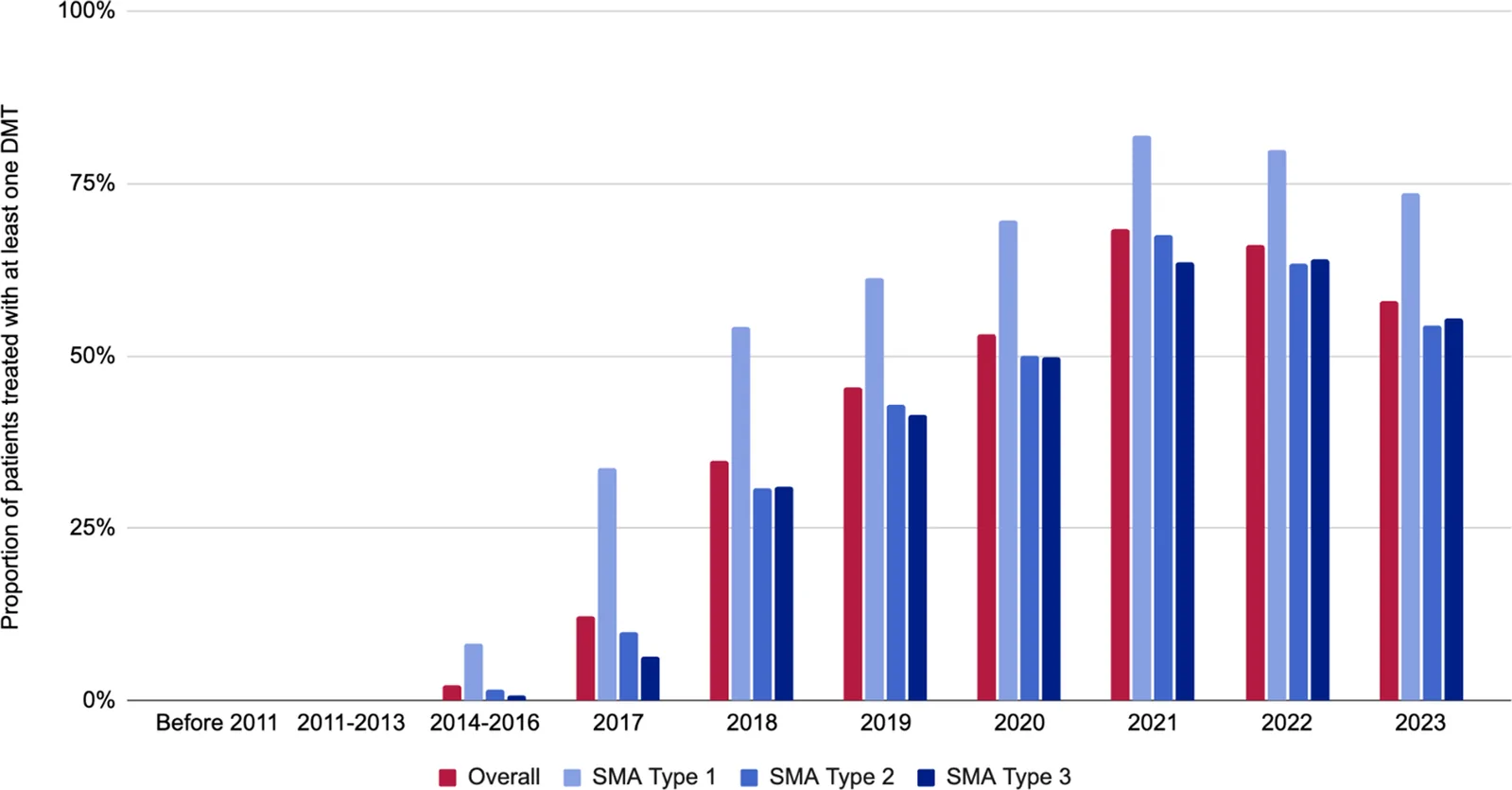
Proportion of patients treated with at least one DMT over time. legends: DMT = disease modifying treatment; SMA = spinal muscular atrophy

The proportion of patients treated increased steadily from 2.2% in 2014–2016 (reflecting participation in clinical trials) to as high as 68.4% in 2021 (reflecting post-market availability of all DMTs). Among 101 patients ever treated with onasemnogene, SMA1 comprised the largest group. The number of patients treated with more than one DMT was low, ranging from fewer than five patients in 2019 to 69 (5.4%) patients in 2022. (Fig. 2; Supplemental Table 1).

The most common best functional status after treatment for patients with SMA1 was “sitter” (36.6%), followed by “non-sitter” (23.6%) and “walker” (12.0%), respectively (Table 4). For SMA2, best functional status was also “sitter” (60.9%), but followed by “walker” and “non-sitter” (5.9%). For SMA3, best functional status was “walker” (87.8%), followed by “sitter” (5.3%), and “non-sitter” (1.5%). The most common best motor milestone after treatment was “Sit without support” for patients with SMA1 (27.2%) and SMA2 (38.0%), followed by “Roll onto side” (18.5%) for SMA1 and “Walk with assistance” (15.9%) and “Sit without support” (13.9%) for SMA2. For SMA3, the best motor milestone was “Climb stairs” (63.4%), followed by “Walk 10 metres without assistance” (19.3%). Missingness of best functional status and motor milestone data was highest among patients with SMA1 (27.9%) and lowest among patients with SMA2 (8.5%) and SMA3 (5.5%). Almost all patients (97.4%) were missing best functional status prior to treatment. Among the never treated patients, best functional status was missing for all patients with SMA1 and SMA3, and almost all patients with SMA2 (97.8%).

**Table 4:** Best achieved functional status, motor milestones, and event-free survival among treated patients, overall and by SMA type

	Overall (*N* = 1321)	SMA Type 1 (N = 276)	SMA Type 2 (N = 540)	SMA Type 3 (N = 476)
**Best functional status before treatment; n (%)**				
Non-sitter	*	*	*	0 (0.0%)
Sitter	15 (1.1%)	0 (0.0%)	15 (2.8%)	0 (0.0%)
Walker	18 (1.4%)	0 (0.0%)	*	14 (2.9%)
Missing	1286 (97.4%)	275 (99.6%)	521 (96.5%)	462 (97.1%)
**Best functional status after treatment; n (%)**				
Non-sitter	106 (8.0%)	65 (23.6%)	32 (5.9%)	7 (1.5%)
Sitter	460 (34.8%)	101 (36.6%)	329 (60.9%)	25 (5.3%)
Walker	600 (45.4%)	33 (12.0%)	133 (24.6%)	418 (87.8%)
Missing	155 (11.7%)	77 (27.9%)	46 (8.5%)	26 (5.5%)
**Best motor milestone after treatment; n (%)**				
Hold head without support	41 (3.1%)	14 (5.1%)	21 (3.9%)	5 (1.1%)
Roll onto side	65 (4.9%)	51 (18.5%)	11 (2.0%)	*
Sit without support	296 (22.4%)	75 (27.2%)	205 (38.0%)	13 (2.7%)
Crawl	92 (7.0%)	6 (2.2%)	75 (13.9%)	9 (1.9%)
Stand with assistance	62 (4.7%)	20 (7.2%)	42 (7.8%)	0 (0.0%)
Stand without assistance	10 (0.8%)	0 (0.0%)	7 (1.3%)	*
Walk with assistance	107 (8.1%)	18 (6.5%)	86 (15.9%)	*
Walk without assistance	48 (3.6%)	8 (2.9%)	16 (3.0%)	22 (4.6%)
Walk 10 metres without assistance	115 (8.7%)	5 (1.8%)	16 (3.0%)	92 (19.3%)
Climb stairs	330 (25.0%)	*	15 (2.8%)	302 (63.4%)
Missing	155 (11.7%)	77 (27.9%)	46 (8.5%)	26 (5.5%)
**Best score for CHOP-INTEND**				
Median [IQR]	46 [33.8, 56.2]	44 [33, 54]	46 [35, 57.5]	51 [28.5, 64]
Missing	1105 (83.6%)	170 (61.6%)	453 (83.9%)	466 (97.9%)
**Best score for HFMS(−E)**				
Median [IQR]	29 [8.2, 50]	15 [3, 27]	12.5 [4, 31.2]	50 [29.8, 58]
Missing	907 (68.7%)	221 (80.1%)	372 (68.9%)	296 (62.2%)
**Best score for RULM**				
Median [IQR]	24 [14, 36]	11.5 [8.2, 19.5]	17 [10, 24]	35.5 [26.8, 37]
Missing	967 (73.2%)	242 (87.7%)	377 (69.8%)	328 (68.9%)
**Event-free survival, months (death or permanent ventilation)**				
Mean (SD)	49.7 (92)	21 (37.9)	180.4 (140.2)	-
Median [IQR]	13 [6, 35]	10 [6, 24]	143 [83, 246]	-
Number (%) of censored patients	1271 (96.2%)	235 (85.1%)	531 (98.3%)	476 (100.0%)

CHOP-INTEND = Children’s Hospital of Philadelphia Infant Test of Neuromuscular Disorders; HFMS(−E) = Hammersmith Functional Motor Scale - Expanded; IQR = interquartile range; RULM = Revised Upper Limb Module; SMA = spinal muscular atrophy

Note: * indicates *n* < 5 patients

Functional SMA statuses correspond to the following best-achieved motor milestones: Non-sitter: patients who can or not “hold head up without support” or “roll onto side.” Sitter: patients who can “sit without support,” “crawl,” “stand with assistance,” “stand without assistance.” Walker: patients who can “walk with assistance, walk without assistance, walk 10 metres without assistance, climb stairs.”

Functional status and motor milestones among the never-treated group were missing in 99.1% of patients overall (100.0% among SMA1, 97.8% among SMA2, 100.0% among SMA3) and thus are not reported. Similarly, scores for CHOP-INTEND, HFMS(-E), and RULM were missing for 97.8%-98.9% of patients overall among the never-treated group

Evaluation by a validated scales was missing in a large majority of patients. CHOP-INTEND, which constitutes the most commonly used scale in SMA1 was available in only 38.4% of patients living with SMA. HFMSE which constitutes the most frequently used scale in patients living with SMA2 was available in only 31.1% of them.

## Discussion

In this multi-registry study conducted among 2,188 patients with genetically confirmed 5q SMA from patient- and clinician-reported registries representing nine countries across Europe, SMA disease progression and treatment with DMTs were described overall and by SMA type over the 15 years represented in these data. While SMA1 is known to be the most common subtype, SMA2 was the most common subtype in our study followed by SMA3 and then SMA1 (41.8%, 35.6%, and 19.7%). SMA1 cases may be under-represented in registries due to insufficient time to register, since the typical life expectancy of SMA1 is two years without treatment and infants with more severe cases may not live long after initial diagnosis. The median ages at symptom onset for SMA1, SMA2, and SMA3 (2 months, 10 months, and 2.7 years) were within typical age ranges of symptom onset [24].

Among those treated in our cohort with SMA1, the most common best-achieved functional status and motor milestone were “sitter” (36.6%) and “sit without support” (27.2%), respectively. These benchmarks are similar to results from an open-label study that reported 29% of infants with SMA1 were able to sit without support after one year of risdiplam treatment [25]. In our cohort, 12.0% of treated patients had “walker” as their best achieved status, which is similar to results from the first clinical trial of onasemnogene where 16.7% of patients with SMA1 treated with a high dose of gene therapy gained the ability to walk [10, 26]. Although our results are similar to these prior studies, direct comparison cannot be made due to the high amount of missingness for functional status in our study (27.9% of patients), differences in length of follow-up and includes more patients identified by newborn screening.

Considerable differences in functional status were observed across registries, which is not surprising considering differences in the distribution of missingness (which ranged from 2.7% to 53.7%) and subtypes. These differences may also be driven by differences in assessment by country. For example, the Spain registry documented the highest percentage of walkers in the treated SMA1 population (27.1%). This cannot be explained by the definition of « walker », as in this study, a ‘walker’ is defined as someone who can or could previously take steps with or without assistance. In contrast RegistrAME considers a walker as someone who can independently walk at least 10 metres at the time of the study, which is more restrictive. Another source of disparity may be due to the definition of patients identified by newborn screening, or due to having an affected sibling. If these patients are treated presymptomatically, it may not be possible to classify patients as type 1, 2 or 3. Nevertheless, they can be sometimes classified based on genotype or phenotype of the affected sibling. This could also go towards explaining the higher frequency of “walkers” amongst SMA1 patients.

SMA classification relies on highest functional milestone achieved without treatment : SMA2 patients can sit independently but not walk, whereas untreated SMA3 patients develop independent walking [2, 4, 5]. This could constitute an easy way to confirm SMA type in the registries. Unfortunately, very few functional status measurements were reported for patients who were never treated in the registries included in this study to allow for the confirmation of SMA type or meaningful comparison between patients group based on highest functional milestone achieved.

There are several strengths and limitations to consider when interpreting the results, and lessons learned for consideration for future studies that wish to compile multiple rare disease registries. The use of registry data presents a unique opportunity for analysis of underrepresented populations who are difficult to recruit for clinical trials given the rarity of SMA.

Despite the rarity of SMA, this large, pan-European study is the first of its kind to pool patients from multiple smaller disease-specific registries to create one large sample with rich data generalisable to European populations and thus valuable for use in European regulatory decision making. Given the number of registries that exist across Europe, conducting a study incorporating these multiple data sources represents a significant effort. Further, these data provided the opportunity to compare patients’ clinical profiles in the context of emerging treatment options and evolving standards in healthcare management. One of the key aspects to consider when interpreting our findings is the nature of the registries themselves – some being clinician-reported, and some being patient-reported for example. This fundamental methodology difference illustrates the broad diversity in registry construction and therefore presented a challenge when combining their results.

Pooling across registries highlighted differences in data collection and the study population for each registry that expanded beyond inherent differences between clinician-reported and patient-reported registries, which may easily be masked in pooled analyses if not also explored at a registry-specific level. Since it is essential to systematically assess the reliability, relevance and accessibility of data sources for use in a regulatory context, the quality and completeness of the registry data for our study were assessed via questionnaires sent to registries that were designed according to EMA and other best-practice guidance [16–19]. The final evaluation of data completeness was subject to each registry owner’s discretion, resulting in variability in quantitative assessment of the data.

Despite this structured and guidance-based process employed, substantial missingness was observed for several variables once the data were obtained for analysis, particularly among never treated patients. For example, over half of those never treated were lost to follow-up, compared with only 5% among those who were treated. This missingness may reflect a less regular and accurate follow-up and/or an underreporting of data in untreated patients, or it could also be that some these patients are misclassified as never treated if they had treatment that is also missing. Higher completion in treated patients may also be attributed to the increasing need for payers to document patient progress with real world data. Nonetheless, the observed lack of data completeness, especially for motor and functional status variables, results in an incomplete picture of this patient population and limits the ability to identify post-diagnostic outcomes of interest or patient prognostic characteristics that may influence treatment decisions. In the future, accurate quantitative assessment of missingness and increased granularity in defining the data requirements should be considered at the feasibility phase of such studies.

An additional challenge was the study period overlapping with the COVID-19 pandemic. COVID-19 was first detected in Europe after the introduction of nusinersen and immediately preceding the introduction of onasemnogene and, as a result, calendar time trends of treatment utilisation may be affected by COVID-19 related impacts to access to care, general strain on healthcare professionals and resources, and deprioritisation of registry data upkeep.

The learnings from the data challenges encountered led to key considerations. First, systematic data quality assurance processes and governance are needed to ensure further development and harmonisation of patient- and clinician-reported registries to enhance their ability to be combined to increase sample size and generalizability, as this would improve the quality of these data for future research and to inform regulatory decisions. In addition, universal standards that simplify outcome measurement could also improve completeness, especially for patient-reported registries. Lastly, while registry datasets have been proposed as natural history data for untreated control arms for drug trials (particularly in the case of rare diseases where a placebo or untreated control group may be neither feasible nor ethical), the results from this study have shown that the high degree of missingness among the untreated patients in both the patient- and clinician-reported registries could prove to be problematic for such applications. Much higher level of funding and oversight of data collections will be needed in order to use current registries as a real external comparator.

## Conclusions

This study demonstrated that the use of multiple federated registries can increase sample size for rare diseases and provide complementary information to address regulatory research questions. While the results among non-missing variables generally aligned with expectations, there was substantial missingness among key variables and differences between registries that cannot be disentangled from the differences in missingness across the registries. An avenue for future research would be to conduct similar exercise with registries that share similar methodological construct (for instance patients-reported Vs HCP reported)

The learnings from the data challenges encountered led to three key considerations: a need for improved processes and governance for communication and collaboration across registries, standards to harmonise and simplify outcome measurement, and that the high degree of missingness among the untreated patients limits the utility of these data to support analysis of untreated control arms for drug trials.

## Electronic supplementary material

Below is the link to the electronic supplementary material.

Supplementary material 1

## Data Availability

The data that support the findings of this study are made available from the TREAT-NMD federated network of registries, but restrictions apply to the availability of these data, which were used under licence for the current study, and so are not publicly available.

## Abbreviations

CHOP-INTEND: Children’s Hospital of Philadelphia Infant Test of Neuromuscular Disorders
ddPCR: Droplet digital polymerase chain reaction
DMT: Disease modifying treatment
EMA: European Medicines Agency
HFMS(−E): Hammersmith Functional Motor Scale - Expanded
HRM: High resolution melting
IQR: Interquartile range
MLPA: Multiplex ligation-dependent probe amplification
NBS: Newborn genetic screening
qRT-PCR: Quantitative real-time reverse-transcription polymerase chain reaction
RFLP: Restriction fragment length polymorphism
RULM: Revised Upper Limb Module
SD: Standard deviation
SMA: Spinal muscular atrophy
SMA1: SMA type 1
SMA2: SMA type 2
SMA3: SMA type 3
SMN1: Survival of motor neuron 1
SPIFD: Structured Process to Identify Fit-For-Purpose Data
UK: United Kingdom
