# Supplementary material for: Use of European registries to characterise the changing landscape of disease progression and treatment of spinal muscular atrophy (SMA): opportunities, pitfalls and challenges
Source: Orphanet J Rare Dis. 2026 Aug 5;21:390. doi: 10.1186/s13023-026-04491-3 (PMC13732853; doi:10.1186/s13023-026-04491-3)
Supplement: Supplementary file 1 [file 13023_2026_4491_MOESM1_ESM.docx]

## Supplemental Results Tables

**Supplemental Table 1. Treatment utilisation over time, overall and by SMA type**

| | Before 2011 | 2011-2013 | 2014-2016 | 2017 | 2018 | 2019 | 2020 | 2021 | 2022 | 2023 |
|---|---|---|---|---|---|---|---|---|---|---|
| Overall (N = 2188 contributing data to at least one calendar year) | | | | | | | | | | |
| Number with available data | 91 | 389 | 872 | 999 | 1408 | 1579 | 1695 | 1496 | 1287 | 783 |
| Treated with at least one DMT; n (%) | 0 (0.0%) | 0 (0.0%) | 19 (2.2%) | 122 (12.2%) | 488 (34.7%) | 717 (45.4%) | 901 (53.2%) | 1023 (68.4%) | 851 (66.1%) | 454 (58.0%) |
| Treated with more than one DMT; n (%) | 0 (0.0%) | 0 (0.0%) | 0 (0.0%) | 0 (0.0%) | 0 (0.0%) | * | 34 (2.0%) | 47 (3.1%) | 69 (5.4%) | 6 (0.8%) |
| Treated with nusinersen; n (%) | 0 (0.0%) | 0 (0.0%) | 19 (2.2%) | 117 (11.7%) | 454 (32.2%) | 669 (42.4%) | 801 (47.3%) | 765 (51.1%) | 547 (42.5%) | 263 (33.6%) |
| Treated with onasemnogene; n (%) | 0 (0.0%) | 0 (0.0%) | 0 (0.0%) | 0 (0.0%) | * | 7 (0.4%) | 29 (1.7%) | 61 (4.1%) | 76 (5.9%) | 42 (5.4%) |
| Treated with risdiplam; n (%) | 0 (0.0%) | 0 (0.0%) | 0 (0.0%) | 5 (0.5%) | 32 (2.3%) | 44 (2.8%) | 105 (6.2%) | 244 (16.3%) | 298 (23.0%) | 155 (19.8%) |
| SMA Type 1 (N = 432 contributing data to at least one calendar year) | | | | | | | | | | |
| Number with available data | 15 | 64 | 147 | 151 | 222 | 263 | 284 | 254 | 239 | 136 |
| Treated with at least one DMT; n (%) | 0 (0.0%) | 0 (0.0%) | 12 (8.2%) | 51 (33.8%) | 120 (54.1%) | 161 (61.2%) | 198 (69.7%) | 208 (81.9%) | 189 (79.8%) | 100 (73.5%) |
| Treated with more than one DMT; n (%) | 0 (0.0%) | 0 (0.0%) | 0 (0.0%) | 0 (0.0%) | 0 (0.0%) | * | 17 (6.0%) | 19 (7.5%) | 24 (10.0%) | 6 (4.4%) |
| Treated with nusinersen; n (%) | 0 (0.0%) | 0 (0.0%) | 12 (8.2%) | 51 (33.8%) | 119 (53.6%) | 158 (60.1%) | 175 (61.6%) | 153 (60.2%) | 109 (45.6%) | 55 (40.4%) |
| Treated with onasemnogene; n (%) | 0 (0.0%) | 0 (0.0%) | 0 (0.0%) | 0 (0.0%) | * | 5 (1.9%) | 21 (7.4%) | 38 (15.0%) | 52 (21.8%) | 26 (19.1%) |
| Treated with risdiplam; n (%) | 0 (0.0%) | 0 (0.0%) | 0 (0.0%) | 0 (0.0%) | 0 (0.0%) | 0 (0.0%) | 19 (6.7%) | 36 (14.2%) | 52 (21.8%) | 25 (18.4%) |
| SMA Type 2 (N = 914 contributing data to at least one calendar year) | | | | | | | | | | |
| Number with available data | 48 | 175 | 395 | 468 | 650 | 709 | 769 | 662 | 562 | 338 |
| Treated with at least one DMT; n (%) | 0 (0.0%) | 0 (0.0%) | 6 (1.5%) | 47 (10.0%) | 200 (30.8%) | 304 (42.9%) | 385 (50.1%) | 447 (67.5%) | 356 (63.3%) | 184 (54.4%) |
| Treated with more than one DMT; n (%) | 0 (0.0%) | 0 (0.0%) | 0 (0.0%) | 0 (0.0%) | 0 (0.0%) | * | 17 (2.2%) | 17 (2.6%) | 20 (3.6%) | 0 (0.0%) |
| Treated with nusinersen; n (%) | 0 (0.0%) | 0 (0.0%) | 6 (1.5%) | 43 (9.2%) | 176 (27.1%) | 271 (38.2%) | 319 (41.5%) | 280 (42.3%) | 202 (35.9%) | 104 (30.8%) |
| Treated with onasemnogene; n (%) | 0 (0.0%) | 0 (0.0%) | 0 (0.0%) | 0 (0.0%) | * | * | 7 (0.9%) | 18 (2.7%) | 16 (2.8%) | 6 (1.8%) |
| Treated with risdiplam; n (%) | 0 (0.0%) | 0 (0.0%) | 0 (0.0%) | * | 23 (3.5%) | 33 (4.7%) | 76 (9.9%) | 166 (25.1%) | 158 (28.1%) | 74 (21.9%) |
| SMA Type 3 (N = 779 contributing data to at least one calendar year) | | | | | | | | | | |
| Number with available data | 23 | 145 | 322 | 373 | 523 | 585 | 619 | 550 | 460 | 285 |
| Treated with at least one DMT; n (%) | 0 (0.0%) | 0 (0.0%) | * | 24 (6.4%) | 162 (31.0%) | 243 (41.5%) | 308 (49.8%) | 350 (63.6%) | 294 (63.9%) | 158 (55.4%) |
| Treated with more than one DMT; n (%) | 0 (0.0%) | 0 (0.0%) | 0 (0.0%) | 0 (0.0%) | 0 (0.0%) | 0 (0.0%) | 0 (0.0%) | 11 (2.0%) | 23 (5.0%) | 0 (0.0%) |
| Treated with nusinersen; n (%) | 0 (0.0%) | 0 (0.0%) | * | 23 (6.2%) | 154 (29.4%) | 234 (40.0%) | 300 (48.5%) | 322 (58.5%) | 231 (50.2%) | 101 (35.4%) |
| Treated with onasemnogene; n (%) | 0 (0.0%) | 0 (0.0%) | 0 (0.0%) | 0 (0.0%) | 0 (0.0%) | 0 (0.0%) | 0 (0.0%) | * | * | * |
| Treated with risdiplam; n (%) | 0 (0.0%) | 0 (0.0%) | 0 (0.0%) | * | 8 (1.5%) | 9 (1.5%) | 8 (1.3%) | 36 (6.5%) | 82 (17.8%) | 53 (18.6%) |

DMT = disease modifying treatment; SMA = spinal muscular atrophy
Note: * indicates n < 5 patients
